# Pop-In Identification in Nanoindentation Curves with Deep Learning Algorithms

**DOI:** 10.3390/ma14227027

**Published:** 2021-11-19

**Authors:** Stephania Kossman, Maxence Bigerelle

**Affiliations:** Laboratoire d’Automatique, de Mécanique et d’Informatique Industrielles et Humaines, LAMIH, Université Polytechnique Hauts-de-France, UMR CNRS 8201, 59300 Valenciennes, France; maxence.bigerelle@uphf.fr

**Keywords:** nanoindentation, pop-in, artificial intelligence, deep learning, computer vision

## Abstract

High–speed nanoindentation rapidly generates large datasets, opening the door for advanced data analysis methods such as the resources available in artificial intelligence. The present study addresses the problem of differentiating load–displacement curves presenting pop-in, slope changes, or instabilities from curves exhibiting a typical loading path in large nanoindentation datasets. Classification of the curves was achieved with a deep learning model, specifically, a convolutional neural network (CNN) model implemented in Python using TensorFlow and Keras libraries. Load–displacement curves (with pop-in and without pop-in) from various materials were input to train and validate the model. The curves were converted into square matrices (50 × 50) and then used as inputs for the CNN model. The model successfully differentiated between pop-in and non-pop-in curves with approximately 93% accuracy in the training and validation datasets, indicating that the risk of overfitting the model was negligible. These results confirmed that artificial intelligence and computer vision models represent a powerful tool for analyzing nanoindentation data.

## 1. Introduction

For decades, instrumented indentation testing, particularly nanoindentation, has been a powerful and practically compulsory technique for the mechanical characterization of materials at nano- and micro-metric scales. Novel approaches to this technique have emerged, such as high–speed indentation. This new method has become very popular in recent years as an advanced tool for the mechanical characterization of complex microstructures and multi-phase materials. The main advantage of this technique is that it can perform hundreds of imprints in a few minutes (or even a few seconds), thus creating mappings of the mechanical properties [1]. It also decreases thermal drift effects [1]. The large datasets obtained using this method can utilize advanced statistical methods and artificial intelligence algorithms [2,3,4]. High–speed indentation is suitable for all materials [5,6], although specifically, for multi-phase materials [7].

When we analyze large nanoindentation datasets (hundreds to thousands of curves), we will not probably focus on detailed analysis of each load–displacement (*P*–*h*) curve. Therefore, the mechanical properties could be miscalculated, particularly if the curves exhibit pop-in events (i.e., abrupt displacement bursts at a constant load in force–control tests, or force drops in displacement–control tests). Pop-in incidents are related to numerous causes, such as cracking, coating delamination or chipping [8,9,10], incipient plasticity, dislocation nucleation, dislocation density [11,12], dislocation avalanches [13], grain boundaries [12,14], shear bands [15], surface roughness [12,16], and phase transformation [17].

Pop-in events (in load–control tests) increase the maximum displacement and the unloading curve’s shape, possibly affecting accurate estimations of the hardness and elastic modulus. Nevertheless, in-depth studies of pop-in events provide information about the mechanical behavior of materials; for example, in brittle materials and coatings, fracture toughness can be assessed [8,9].

On the one hand, many authors have addressed the problem of pop-in identification, using different existing methodologies to detect and quantify pop-in events. For example, Malzbender and de With [18] used the plots of *P/h*^2^ vs. *h*^2^ to detect slope changes and the derivative d*P*/d*h*^2^ vs. *h*^2^ to identify local minimums. Juliano et al. [19] also implemented a method based on the derivative d*P*/d*h* at a given *h*_x_. Sato et al. [20] set a threshold value above which the displacement burst is considered a pop-in for a given acceptable load change (ideally, about zero). Similarly, Bolin et al. [21] studied pop-in event probability by setting a threshold on the displacement differences equal to the machine noise threshold. Data smoothing can be necessary before differentiation, as addressed by the authors in [18,22]. Recently, Mercier [23] developed a toolbox for pop-in detection based on some of the above methodologies and a function for peak detection. These methods are mainly used in load–control tests.

On the other hand, pop-in identification is not necessarily a straightforward process. A simple illustration is a discrimination between electrical and mechanical noise from a pop-in event [20], which is also affected by the sensors resolution and data acquisition rate, e.g., sliding pop-in [8]. The latest nanoindentation instruments have high–resolution sensors, enabling rapid data acquisition rates and time constants. In this kind of instrument, typical pop-in displacement bursts might be overlooked [24]. Therefore, the described methodologies for pop-in detection may be impractical. Phani and Oliver [24] recently demonstrated that the response of pop-in events occurs at the microsecond (µs) scale, measurable with state-of-the-art instruments. In contrast, most reported data on pop-in behavior have been obtained on instruments with a time constant in the order of milliseconds, losing important information about pop-in events. A strong dependence between the pop-in length and the actuator mass has also been found.

Hardness and elastic modulus calculations by nanoindentation require load–displacement curves free of pop-in events. This work introduces the use of a deep learning model (convolutional neural network (CNN)) to classify load–displacement curves. The classification is based on the presence of pop-in, sub-pop-in, instability step, and slope change events from those that present a typical loading path in load-control tests. This study aims to provide the first methodology for *P*–*h* curve classification in large nanoindentation datasets. The possibility of identifying anomalies in the curves could provide additional information about the mechanical behavior of materials.

The implementation of artificial intelligence (machine learning, neural networks, deep learning, etc.) to analyze nanoindentation data has emerged in the past few years. This area has studied different subjects, such as the characterization of heterogeneous materials to differentiate between constituent phases [25,26,27]; the identification of stress–strain curves [28,29]; and the study of acoustic emission events induced by nanoindentation [30]. However, for decades, the combination of nanoindentation and artificial intelligence has been used to identify viscoelastic parameters [31] and Poisson’s ratio [32,33] using neural networks. Numerous interesting approaches have resulted from the combination of both fields, given more powerful resources for understanding the mechanical behavior of materials.

## 2. Methods

### 2.1. Nanoindentation Testing

The nanoindentation datasets used to perform this investigation were obtained using a TriboIndenter TI-980 (Hysitron^®^, Bruker, Minneapolis, MN, USA) [34]. All the tests were carried out with a Berkovich diamond tip at controlled room temperature (~20 °C). The tests were performed in accelerated indentation mode (XPM) under load control.

The dataset included different materials, such as ceramic coatings, aluminum alloys, fused quartz, and silicon. The maximum applied load and displacement were below 3 mN and 1 µm, respectively; the loading rates ranged between 500 and 1250 µN/s. The dataset was composed of 744 load–displacement (*P*–*h)* curves. The curves were individually plotted and visually classified between curves presenting pop-ins and slope changes (342 curves) from those presenting a regular loading path (402 curves). The mechanical properties of the studied materials varied between 1 and 10 GPa for hardness and between 65 and 170 GPa for elastic modulus.

### 2.2. Data Preparation

**Figure 1:**
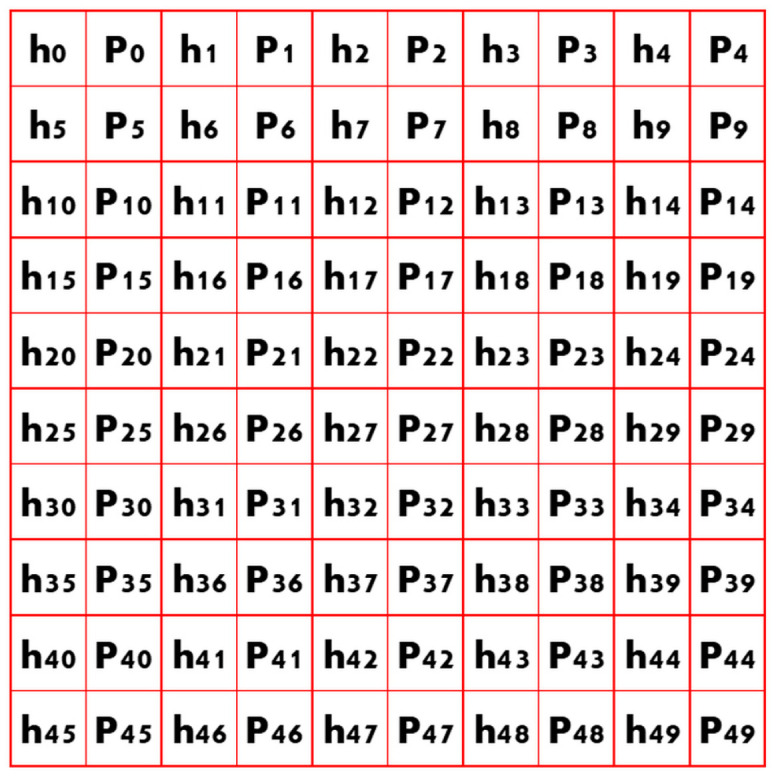
Example of a matrix constructed from the load–displacement curve dataset, here assuming a dataset with 50 rows and 2 columns (*h, P*) arranged in a matrix of float 10 × 10.

The load–displacement curves data files had 500 or 1200 data rows for each column (displacement (*h*) in nm and load (*P*) in µN). Each load–displacement curve dataset (loading-hold-unloading) was converted (reshaped) into a matrix of dimensions equal to 50 × 50 (rows × columns), schematically represented in Figure 1. The dimensions were selected as a function of the maximum number of data points (2400) in the load–displacement curves (1200 rows × 2 columns). The rest of the matrix was filled through a zero-padding technique to build matrices of the same size.

All the matrices were organized in a dataset *X* (independent variable). Then, the dataset was scaling, its minimum value was subtracted, and then it was divided by the difference between the maximum and minimum values of dataset *X* (this operation is known as MinMaxScaler in the scikit-learn library). The objective of scaling the data was to reduce the asymmetry of the multiple dimensions of data (*h*, *P*), helping with the training process of the CNN model, explained in the next section.

We set a Boolean variable *Y* (1 or 0) for each matrix, where 1 and 0 represented the existence or absence of pop-in (or slope change) events in the load–displacement curves, respectively; a total of 342 matrices were assigned a value of 1 (pop-in), and 402 were assigned a value of 0 (no pop-in).

#### Cross-Validation of the Data

The dataset was split into a 70/30 ratio, of which 70% (520 matrices) was used to train the model, as described in the next section, and 30% (224 matrices) was used to perform the validation test. The split of the data consisted of a random picking of the numbers without replacement.

### 2.3. Convolutional Neural Networks (CNN) Model

**Figure 2:**
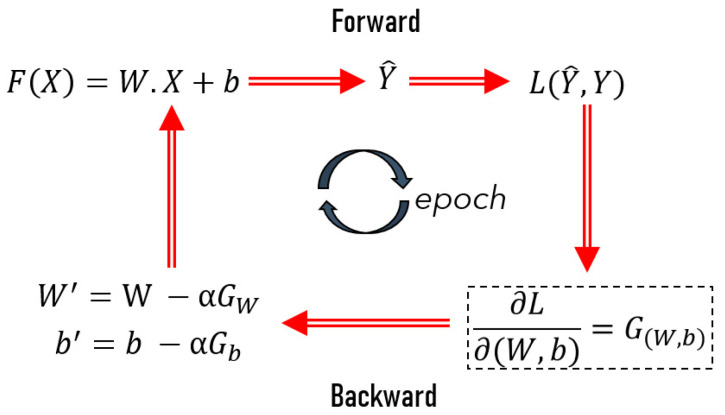
The overall workflow of a CNN.

CNNs are deep neural networks specialized in image recognition, and imitate how the visual cortex of the brain processes and recognizes images [35]. The load–displacement data were transformed into matrices to apply this type of algorithm to classify the load–displacement curves. An overall workflow of how CNNs work is schematized in Figure 2.

As illustrated in Figure 2, the general training procedure of a CNN model is decomposed into the iterative and successive application of two main steps, forward and backward propagation, summarized in the following phases:

1. In the forward propagation: a function, *F*, is dependent on the input variable *X* (e.g., images), a given number of linear operators *W* (filters or image kernels), and multiple bias terms (*b*) are applied to estimate a prediction output $\hat{Y}$.
2. In backpropagation:
  a. The loss function, *L*, is used to calculate the difference between the generated output $\hat{Y}$ and the expected output Y (given by the data);
  b. Then, the gradient, G, (partial derivatives of *L* with respect to *W* and *b*) of the loss function is calculated to update the values of the filters and bias terms ($W^{\prime },b^{\prime })$.
3. The updated values of the filters and bias terms are introduced in step *i*, repeating the forward and backward propagation of a defined number of iterations (epochs) until approaching the minimum of the loss function.

In summary, the main objective of the training process is to find out the best values for *W* and *b* [35].

In this study, a CNN model was built using the open-source libraries TensorFlow 2.0 and Keras. The CNN was composed of three convolutional layers. We applied the rectified linear activation function (ReLu), followed by a pooling layer for each layer. Then, a full connection step was set, composed of three dense, fully connected layers and two dropout layers in between to avoid overfitting. The functions applied to these layers were ReLu and Sigmoid.

The selected loss function corresponded to the calculation of the binary cross-entropy [36], which is suitable for the binary classification problem (pop-in or not pop-in). For the descendent gradient optimization of the CNN, the optimizer “Adam” was implemented [36].

The model iterations were set to an early stopping criterion, which consisted of stopping the model’s training process if, after 20 epochs, there was no improvement in the validation loss.

**Figure 3:**
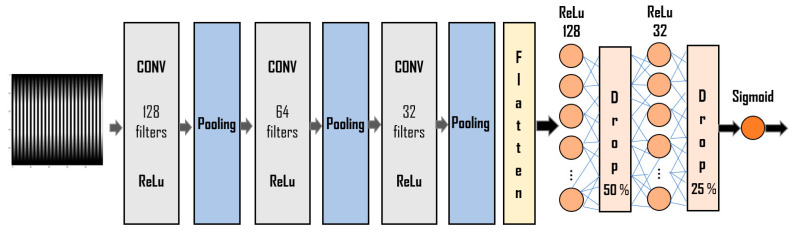
The architecture of the CNN model implemented to classify curves with pop-in and without pop-in events.

The schematic representation of the CNN architecture is given in Figure 3. The detailed code was implemented in the open-source programming language Python 3.7.

## 3. Results and Discussion

### 3.1. Convolutional Neural Network (CNN) Model

**Figure 4:**
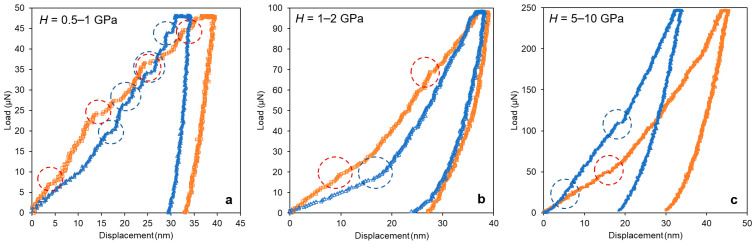
Load–displacement curves (load–control tests) showing pop-in events and the range of hardness (*H*) values. (**a**) sub-pop-ins in Al alloys; (**b**) slope changes and sliding pop-ins in Al alloys; (**c**) slope changes and pop-ins in ceramic coatings. In each plot, each curve represents a different material that exhibits similar pop-in event features.

Figure 4 shows six examples of *P*–*h* curves, which correspond to different materials exhibiting various pop-in events, such as sub-pop-ins (small pop-ins at shallow penetration depths), sliding pop-ins, and slope changes.

**Figure 5:**
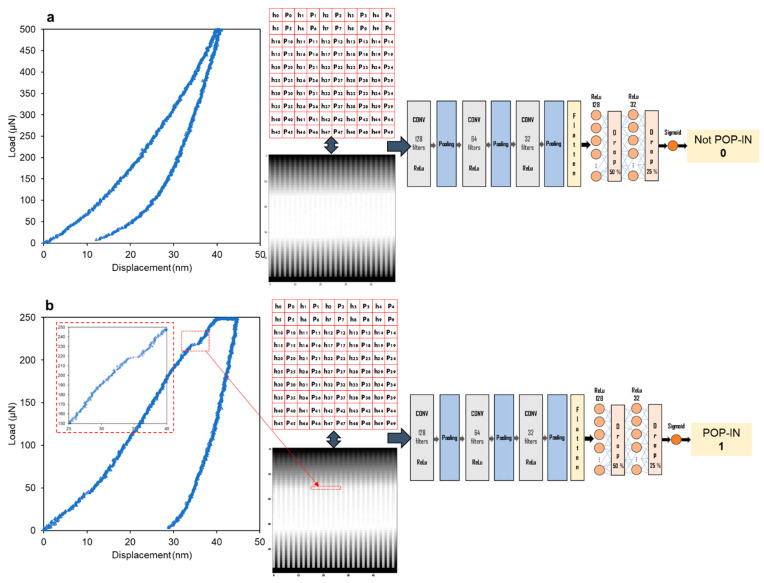
CNN implementation workflow: load–displacement curves (**a**. typical loading path, **b**. pop-in) are converted into matrices (“images” in grayscale) which are the inputs to pass to the CNN model, which give an output equal to **0** for no pop-in and **1** for pop-in events. The numerical matrices are just for illustration and do not represent the model’s 50 × 50 matrices used as input.

Figure 5 presents the schematic workflow of CNN implementation for two curves, presenting a typical loading path and pop-in and their corresponding escalated matrix generated from the load–displacement data.

**Figure 6:**
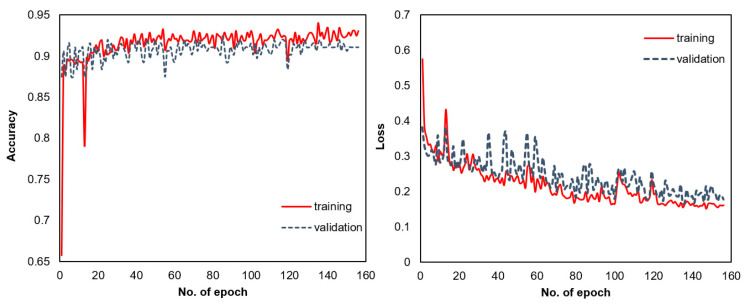
Evolution of the accuracy and loss function during the training and validation of the CNN model as a function of the number of epochs.

The CNN model was fully trained after 158 epochs (the early stopping criterion stopped the training), as illustrated by the evolution of the values of the loss function and the accuracy (Figure 6).

The fully trained CNN model achieved 93% accuracy and a 0.16 binary cross-entropy value on the training dataset (i.e., the lower this value, the better the model). For the validation dataset, a 91% accuracy and 0.17 binary cross-entropy value were achieved. Comparing these performance parameters between the training and validation datasets confirmed that the risk of overfitting was negligible. It is worth mentioning that the model’s training process was executed at least three times, leading to similar accuracy values between 91% and 93%. The average training time of the model was 83 s, running on a GPU (NVIDIA Tesla K80 GPU) available for free and provided by Google Colaboratory.

Table 1 shows the summary (precision, recall, and F-1 score) of the model’s performance in the validation dataset.

**Figure 7:**
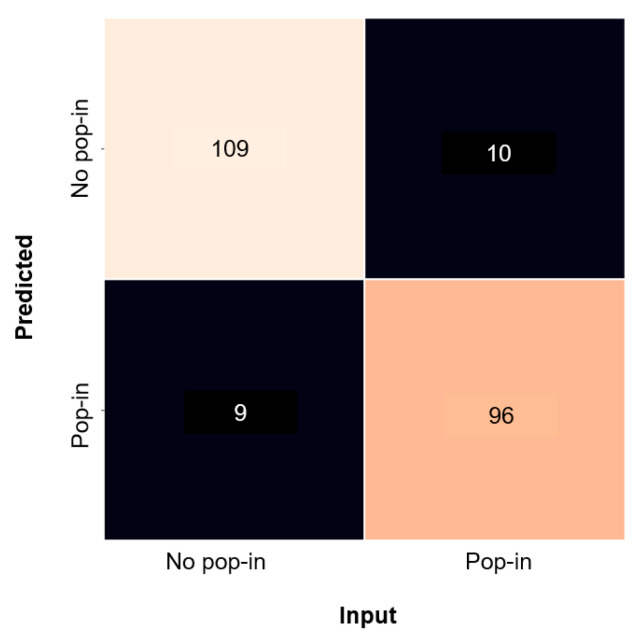
Confusion matrix obtained from the validation dataset tested in the CNN model.

The recall, precision, and F1-score values demonstrated that the model was well balanced, given a similar performance when classifying between pop-in matrices and no pop-in matrices. Nevertheless, the model showed slightly better performance (2%) for the classification of curves without pop-in events, as illustrated in the confusion matrix (Figure 7). This trend was expected because typical nanoindentation load–displacement curves are easier to identify. All the curves presented in Figure 4 were successfully classified as pop-in, i.e., curves presenting sub-sequent sub-pop-ins (Figure 4a) were easier to identify with the model.

### 3.2. Robustness Evaluation of the CNN Architecture and Model

We evaluated the robustness of both the CNN architecture and model, to differentiate between curves presenting pop-in and those without pop-in events with two approaches, as described below.

#### 3.2.1. Influence of the Unloading Curve on the Accuracy of the CNN Model

The CNN architecture presented in Figure 3 was trained and validated using a dataset corresponding to the loading segment of the load–displacement curves.

The resulting model had an accuracy of 92% in the validation dataset, similar to the results presented in Section 3.1., where the model was evaluated using all the load–displacement curves. The summary of the performance of the model in the validation dataset is presented in Table 2. These results corroborate that the implemented CNN architecture correctly identified the features corresponding to pop-in events as relevant information to differentiate the load–displacement curves, and that the unloading curves did not have a major effect.

#### 3.2.2. Artificial Pop-ins

**Figure 8:**
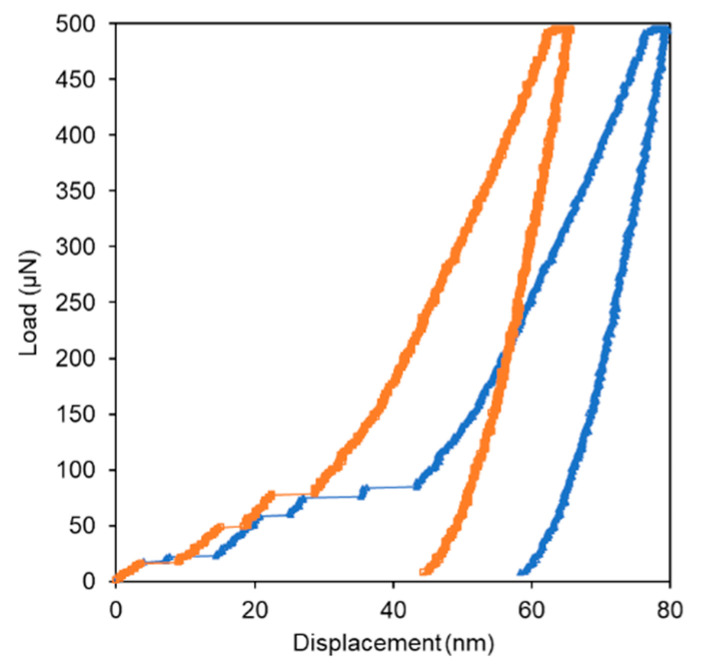
Load–displacement curves showing artificial pop-ins generated at two different times for the same curve.

In order to test the performance of our model to detect subsequent sub-pop-ins, we generated a new dataset with artificial pop-ins. These artificial pop-ins represented gaps in the displacement data of the loading curves; they were generated in the dataset without pop-ins. Figure 8 shows an example of the *P*–*h* curve with artificial pop-ins.

The pop-in lengths were randomly assigned from 2 to 10 nm, and located up to 50% of the maximum displacement. The probability of a pop-in occurring was set to 0.5. This procedure was repeated 10 times for each curve.

This new dataset with artificial pop-ins was tested in our model, obtaining an accuracy of approximately 70%. In this case, these data were not used to train the CNN; only to test the model.

Next, we introduced a dataset with artificial pop-in events as part of the dataset with real pop-ins to train and validate the model, following the same procedure as described in Section 2.2 and Section 2.3. The accuracy of the model was 93%. The performance of the model in the validation dataset is presented in Table 3.

After generating the new model, we tested a new dataset with artificial pop-ins (different from the one used to train the model because pop-in length, occurrence, and location were randomly selected: Figure 8). In this scenario, the model classified the artificial pop-ins with 96% accuracy.

These results highlighted apparent differences in the model performance when only the testing dataset with artificial pop-ins or the artificial pop-ins were included as part of the dataset to train the CNN (70 vs. 93%). This output indicated that the pop-ins in our initial experimental data represent complex features beyond displacement bursts. Thus, the model could detect these complex pop-ins events; additionally, it probably considered relevant information around the pop-in events.

The robustness analysis corroborated that the selected architecture of the CNN is suitable to differentiate curves with pop-ins. This classification is crucial for accurate estimation of the mechanical properties by indentation, especially when dealing with large datasets (hundreds to thousands of tests).

We want to highlight that the overall accuracy of the model (~93%) could have been improve by employing a larger dataset and using more materials. In addition, methodologies related to the treatment of the data, such as noise analysis (reduction or inclusion) [38,39], resampling and smoothing [40], and padding configurations [41] could have enhanced the model’s accuracy.

Our study aims to accentuate the importance of combining artificial intelligence and nanoindentation testing for new applications. Our work contributes that of previous investigations. These studies addressed different problems, such as the identification of phases in multi-phase materials and support vector machine classification algorithms [25]; the solutions to inverse problems through a combination of FEM simulations [28], material compositions [42] with experimental nanoindentation/hardness results and neural networks; and the combination of acoustic emission and nanoindentation data with deep learning algorithms [30].

The applications for the combination of nanoindentation datasets and artificial intelligence models are countless. A similar approach to the one investigated in this work could be implemented in reverse engineering and property prediction applications, e.g., abrasion resistance, fracture toughness, the identification between brittle and ductile materials, etc.

## 4. Conclusions

In this study, we constructed a binary classifier based on image detection, which accurately distinguished between the existence or absence of pop-in events in *P*–*h* curves from nanoindentation tests.

The load–displacement data transformation into matrices of the desired size (50 × 50) represented a suitable input to train the CNN model for the dataset.

The dataset of generated matrices was effectively used to train the created CNN model to classify the initial curves with pop-in (sub-pop-ins, slope changes, and instabilities) and without pop-in events. The model achieved 93% accuracy in the classification. Hence, the proposed CNN model architecture was appropriate for effectively sorting the *P*–*h* curves, corroborating the relevance of applying these methods in nanoindentation data analysis.

Similar variation of the accuracy and loss function values on the training and testing datasets of the CNN model confirmed that the risk of overfitting was unimportant.

The robustness analysis of the CNN architecture revealed two critical aspects. First, evaluation of the CNN model using only the loading curves suggested that this CNN model considered the most relevant information from the loading curves. This information indicated that the model did not seem to be significantly affected by the information contained in the unloading curves. Secondly, through the analysis of *P*–*h* curves with artificial pop-ins in the CNN model, we corroborated the model’s performance to detect pop-in features beyond typical displacement bursts. Both robustness tests corroborated that the selected CNN architecture was suitable to discriminate load–displacement curves with or without pop-in events.

## 5. Suggestions for Future Development

This study opens the possibility for various prospects:

- The quantification of pop-in events (location, length, and probability), which requires an algorithm designed for object detection, a more complex architecture of the neural network, and a different dataset structure. Similarly, the detailed identification of the spatial location of tests where pop-in events occur, pop-in quantification, and their correlation with the mechanical properties by indentation could help better understand the mechanisms which create the pop-ins;
- Studying the effect of aspects related to data configuration (sampling, noise, size, and padding) in the implementation and output of the CNN model;
- Applications of similar models to nanoindentation datasets, including load, displacement, and time variables; additionally, datasets obtained by CSM (continuous stiffness measurement) methods could certainly provide relevant information;
- Applications of similar algorithms to study property prediction, which is an exciting possibility, to establish relationships between nanoindentation data and mechanical properties, e.g., abrasion resistance.

## Figures and Tables

**Table 1:** Summary of the performance of the CNN model in the validation dataset.

Classes	Precision	Recall	F1-Score	No. of Matrices
0 (no pop-in)	0.92	0.92	0.92	119
1 (pop-in)	0.90	0.90	0.90	105

Precision = TP/(TP + FP); Recall = TP/(TP + FN); F1-score = 2 × Precision × Recall/(Precision + Recall); TP: true positive; FP: false positive; FN: false negative [37].

**Table 2:** Summary of the performance of the CNN model in the validation dataset only using the loading curves.

Classes	Precision	Recall	F1-Score	No. of Matrices
0 (no pop-in)	0.94	0.90	0.92	119
1 (pop-in)	0.89	0.93	0.91	105

**Table 3:** Summary of the performance of the CNN model in the validation dataset, including the artificial pop-in dataset in the dataset with pop-ins.

Classes	Precision	Recall	F1-Score	No. of Matrices
0 (no pop-in)	0.93	0.87	0.90	116
1 (pop-in)	0.94	0.96	0.95	228

## Data Availability

The data and code presented in this study are available on request from the corresponding author. The data are not publicly available because they are part of ongoing studies.
